# A Few Thoughts Suggested by the Present Phase of Medical Skepticism

**Published:** 1858-12

**Authors:** Jas. W. Hughes

**Affiliations:** Berlin Centre, Ohio


*A few Thoughts suggested by the present phase of Medical Skepticism.*
By JAS. W. HUGHES, M. D., Berlin Centre, Ohio.

That ignorant and undisciplined minds should vibrate like the pendulum from one extreme to another, ever passing beyond the truth, ought, perhaps, to awaken no surprise. But, that men of intellect, and erudition, accustomed to reason and investigate, instead of sounding the channel of truth, should alternately veer towards the Scylla or Charybdis of error, as fashion or fancy fills their sails, is certainly strange.

The age is one of skepticism. In science as well as religion, men have grown "wise above what is written," and forgetting that truth is older than error, seem disposed to cast aside, with curling lip and arching brow, as mouldy relics of ignorance and superstition, whatever wears not the verdancy of juvenility. The safe, tried, and steady light of experience with its accumulated facts, is discarded for the ignis fatuus glare of wild, and startling speculations, hatched in the unbalanced, but prolific brain of visionary progressionists.

The science of medicine has not escaped the general onslaught.—Turning with inconsiderate and reckless haste, from a blind and bigoted faith in the efficacy of drugs, to cure all the *ails* that "flesh is, heir to," restless theorists have embraced dark and cheerless expectancy, and are seeking to convert the active and hope-inspiring practice of medicine, into a sad and gloomy "meditation on death." That an unreasonable reliance may be placed on drugs, to the exclusion of other and important means, is doubtless true. Salutary and rational effort may be paralyzed by medical skepticism, is not less true.

Grave and gray-beard Professors, with more of *theory* than *practice*, may reiterate the stale witticism of lying quackery, and enumerate "Doctors" amongst "leading causes of death" in certain diseases.—But the great mass of intelligent, observing, and laboring physicians, will continue to feel that they are not worse than uselss intruders in the chambers of the sick; but honored, and blessed instruments of good, in the amelioration of suffering, and the restoration of health to their afflicted fellow men.

That nature does much in the removal of disease is granted. That many cases of threatening aspect would ultimately recover, if left to themselves is not questioned. But it is a question, whether every case of serious disease, not necessarily fatal, may not be materially benefitted by judicious medication, not excepting the so styled "self-limited" diseases, as the exanthemata, for instance; including typhoid fever.

Much of the suffering, and danger, and I may add duration of those affections, beyond a certain period, is owing to accidental causes, such as local congestions, inflammations, and of sequelæ, that might generally be averted, or removed by proper treatment. Much as has been said of scarlatina, and justly, as a reckless and empirical system of drugging has been condemned in that affection, the doctrine that sees in the burning skin—the sloughing tonsils—and wasting diarrhœa, that attend its severer forms, but so many sickly efforts of nature, to throw off an offending virus, and patiently looks on, an inactive and inefficient observer, is not less reprehensible.

If an experience of more than twenty-five years in the profession, during which time several severe epidemics, and frequent slighter ones have come under the writer's notice, entitles; his opinion to any attention, he records it unhesitatingly; that scarlatina is as amenable to treatment as any other disease of equal severity. If the physician would treat it successfully, instead of wasting time in criminal inactivity, mistaking nature's work of disorganization for a curative process; let him meet the indications as they are developed. Treat the symptoms, not the name, and his experience will be an exception, if the result is not satisfactory.

Equalize the circulation and remove congestion in the outset by an emetic of ipecac, aided if necessary by warm effusions and sinapisms; or if the skin be hot, and excitement high, control it with verat. vir.: remove offensive secretions by small doses of rhei. or ol. Ric: or if diarrhœa attend, restrain it with chalk, jalap, astringents, or pulv. doveri.; correct tendency to putrescency, by a free use of chlorate of potash; excite the kidneys to action, by spts. nit. dulc. in some diuretic tisan; *keep the throat as clean as possible, by frequently "swabbing" it with acidulated, antiseptic, or stimulating gargles* as the type may indicate, *applying* occasionally to the *tonsils* and *fauces*, a strong solution of *nit. argt.* to excite a new action in the part.

If collapse approach, camph. and carb ammo. wine, quinine, and capsicum, of course will be our remidies. Bearing in mind the peculiar type of the prevailing epidemic, and adopting our remedies to the abnormal deviations of each particular case, we shall obtain results satisfactory to ourselves, and honorable to an enlightened and philanthropic profession.—*Cincinnati Lancet & Observer.*

